# The 2018 PhD Grant Recipients of the BSR

**DOI:** 10.5334/jbsr.1682

**Published:** 2018-11-17

**Authors:** Alain Nchimi

**Affiliations:** 1 University of Liège and Université Libre de Bruxelles, BE

In 2017–2018, the Belgian Society of Radiology (BSR) continued its efforts to close the gaps of knowledge and disseminate new concepts in radiology. This year’s special number of the *Journal of the Belgian Society of Radiology* (*JBSR*), issued for the annual symposium of the BSR, again honors our PhD grant recipients. These highly graduated individuals present themselves and summarize their long-standing work and academic careers. In this article the summaries of their doctoral theses are available on the site of the *JBSR*: https://www.jbsr.be/.

**Figure:**
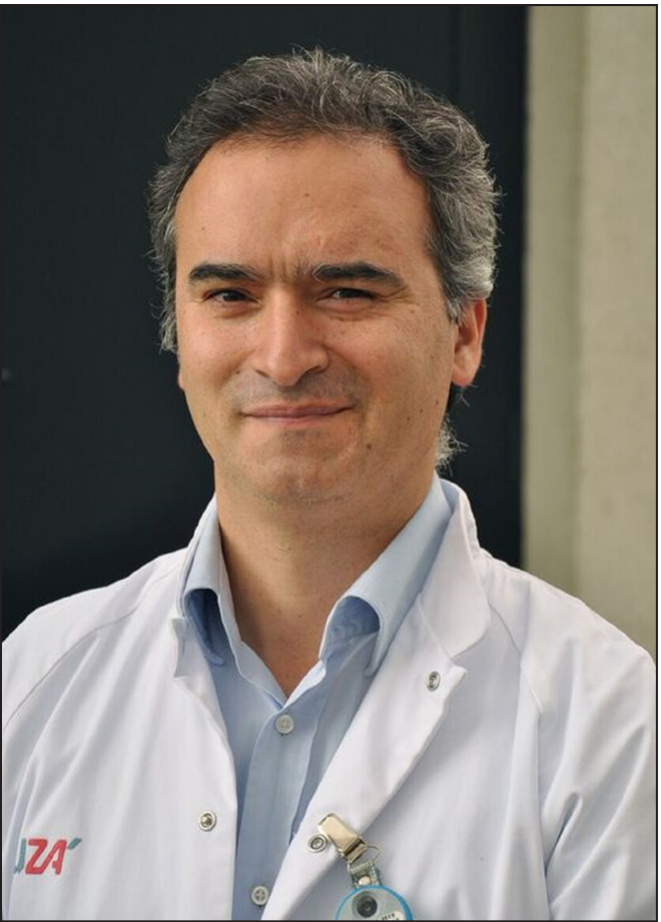
Dr Rodrigo Salgado (2017).

Rodrigo Salgado was born on December 8, 1971, in Independencia (Santiago), Chile. After secondary school, he graduated in 1993 with a degree in computer engineering. That same year, he started his medical studies at the University of Antwerp, graduating in 2000 (cum laude). From 2000 to 2005 he fulfilled his radiology residency under supervision of Prof. Dr. A. De Schepper and Prof. Dr. P.M. Parizel at the Antwerp University Hospital.

After his residency, he achieved a staff position in 2005 at the radiology department of the University Hospital Antwerp, chaired by Prof. Dr. P.M. Parizel. During the following decade, and continuing through the present, he specialized in non-invasive cardiovascular imaging using CT and MR. Currently, he’s a consultant radiologist at the Antwerp University Hospital and a staff member at Heilig Hart Hospital Lier.

Dr. Salgado is an active member of the Belgian Society of Radiology (BSR), the European Society of Radiology (ESR), and the European Society of Cardiovascular Radiology (ESCR). He is currently the elected president of the non-invasive cardiovascular imaging section of the BSR and member of the executive board of the ESCR, in which he is also chair of the Communication and New Media committee. He is member of the European Congress of Radiology (ECR) 2018, 2019, and 2020 scientific subcommittees for cardiac imaging, and serves on the scientific editorial board of *Insights into Imaging* and the *International Journal of Cardiovascular Imaging*. In the past, he has been a member of both the executive and scientific board of the BSR. He is the editor of the ESR publication on the International Day of Radiology 2018 (Cardiac Imaging), and congress president of the ESCR 2019 Congress which will be held in Antwerp, 24–26 October 2019. Dr Salgado presented and defended an original PhD thesis in 2017 at the Antwerp University entitled, ‘Non-invasive Imaging for Cardiovascular Interventions: An Evolving Paradigm’.

**Figure:**
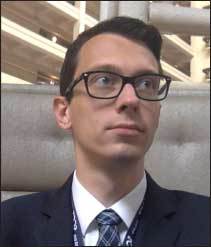
Dr Rolf Symons (2018).

Rolf Symons completed his medical degree at the KU Leuven in 2013 (highest distinction and felicitations of the jury). During his residency in radiology, he completed his PhD in 2018 in a joint project between the University Hospitals Leuven and the National Institutes of Health (NIH)/Johns Hopkins University (JHU) for a dissertation entitled ‘The Role Of State-of-the-Art and Emerging Non-Invasive Imaging Techniques in Improving Risk Stratification in Patients with Coronary Artery Disease’ with promotor Jan Bogaert, MD, PhD, and David A. Bluemke, MD, PhD. He authored 28 publications in international peer-reviewed journals and two book chapters with more than 30 presentations at international conferences, including RSNA 2016, 2017, and 2018. He is an active member of the Radiological Society of North America (RSNA), Society of Cardiovascular Computed Tomography (SCCT), and Belgian Society of Radiology (BSR) and a reviewer for numerous journals including *Radiology, Investigative Radiology, European Radiology, European Heart Journal, JACC Cardiovascular Imaging*, and the *International Journal of Cardiovascular Imaging*. He won the Young Investigator Award (YIA) at the Society of Cardiovascular Computed Tomography (SCCT) 2016 conference, Orlando, Florida (2016), with a presentation entitled ‘CCTA Reproducibility of Coronary Plaque Volume: Sample Size Implications for Clinical Trials’. He has been awarded an NIH and Research Foundation Flanders (FWO) grant for his research. The goal of his research is to use state-of-the-art cardiovascular magnetic resonance (CMR) and emerging cardiovascular computed tomography (CCT) technology to elucidate the relationship between epicardial coronary flow, microvascular dysfunction, clinical risk factors, and adverse left ventricular (LV) remodeling in patients with coronary artery disease (CAD). The unique tissue characterization capabilities of CMR allow for a non-invasive assessment of microvascular obstruction (MVO) and intramyocardial hemorrhage (IMH), two markers of microvascular damage. These CMR markers could be used to improve the short- and long-term prognostication of patients after myocardial infarction. Second, emerging spectral dual-energy and photon-counting CCT technology may allow for a more detailed and reproducible assessment of both epicardial blood flow and myocardial perfusion due to a substantial improvement in tissue characterization capabilities, lower image noise, and higher spatial resolution.

